# Daily measurement of slow slip from low-frequency earthquakes is consistent with ordinary earthquake scaling

**DOI:** 10.1126/sciadv.aaw9386

**Published:** 2019-10-02

**Authors:** William B. Frank, Emily E. Brodsky

**Affiliations:** 1 Department of Earth Sciences, University of Southern California, Los Angeles, CA, USA.; 2 Department of Earth and Planetary Sciences, University of California, Santa Cruz, Santa Cruz, CA, USA.

## Abstract

Slow slip transients on faults can last from seconds to months and stitch together the earthquake cycle. However, no single geophysical instrument is able to observe the full range of slow slip because of bandwidth limitations. Here, we connect seismic and geodetic data from the Mexican subduction zone to explore an instrumental blind spot. We establish a calibration of the daily median amplitude of the seismically recorded low-frequency earthquakes to the daily geodetically recorded moment rate of previously established slow slip events. This calibration allows us to use the precise evolution of low-frequency earthquake activity to quantitatively measure the moment of smaller, subdaily slip events that are unresolvable by geodesy alone. The resulting inferred slow slip moments scale with duration and inter-event time like ordinary earthquakes. These new quantifications help connect slow and fast events in a broad spectrum of transient slip and suggest that slow slip events behave much like ordinary earthquakes.

## Teaser

Daily *M* > 4.5 slow transients on tectonic plate boundaries are similar to fast earthquakes.

## INTRODUCTION

Slow slip is a common feature of some of Earth’s most dangerous subduction zones (*1*). These gradual transients are distinct from ordinary earthquakes and demonstrate that plate motion between major earthquakes is not as steady as previously thought, but is rather composed of a rich spectrum of events. A complete continuum of slip modes has been speculated to exist, with event durations from a fraction of a second to many months (*2*). Geodetic observations of slow slip on plate boundaries span years with continuous GPS records, but episodic slip on shorter time scales is largely invisible with standard geodetic techniques. On time scales of several minutes to hours, tremor (*3*) accompanies slow slip and contains seconds-long repetitive earthquakes called low-frequency earthquakes (LFEs) that are indicative of record transient slip (*4*, *5*). What is missing is a quantitative record of motion between the seismic and geodetically recorded events. Can slow slip occur on minute to hour time scales, and if so, how does its behavior compare to previously established relationships for better studied, ordinary earthquakes?

Here, we present a calibration between seismic and geodetic data recorded above a subduction zone and use it to demonstrate that slow slip occurs every day on this plate boundary. We analyze a 2.5-year time period in the Guerrero (Mexico) segment that contains millions of seismically detected LFEs (*6*) and eight geodetically constrained slow slip events of different sizes (*7*, *8*). We first exploit LFE amplitudes to identify otherwise-undetectable slow transients and measure their seismic moment rates ${\dot{M}}_{o}^{\text{seis}}$ during slow slip. We use the term slow transient here to refer to slow slip that occurs on all time scales including those shorter than the sampling rate of the GPS record. The amplitude-based seismic moment rates are then related to the geodetic moment rate ${\dot{M}}_{o}^{\text{geo}}$ of slow slip measured via GPS. We finally use this relationship to turn a catalog of LFE into a catalog of aseismic slow slip transients that reveals daily moment release and constrains scaling relationships between duration, inter-event time, and moment.

## OBSERVATIONS

### Seismic moment rate of LFEs during slow slip

Earthquake displacement amplitudes in the farfield are proportional to the seismic moment rate ${\dot{M}}_{o}^{\text{seis}}$ of the LFE source (*9*). Therefore, we begin the calibration between seismic and geodetic data by measuring the root mean square (RMS) amplitude in a short window centered on the *S* wave and correcting for geometric spreading (see Materials and Methods). The resulting amplitudes vary over four orders of magnitude (fig. S1).

**Fig. 1:**
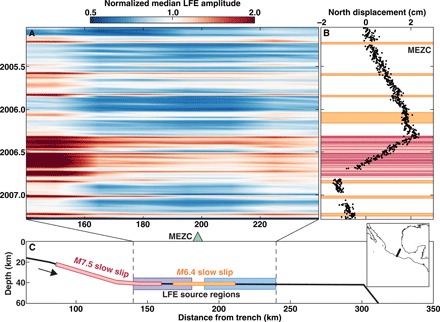
LFE amplitudes track slow slip in both time and space in Guerrero, Mexico. (**A**) Median *S*-wave LFE displacement amplitude in 7-week time bins and 15-km spatial bins, normalized by the mean of all plotted median amplitudes; color scale is logarithmic. (**B**) North-south surface displacement observed at the MEZC GPS station during the 2.5-year LFE record. Colored patches mark geodetically observed slow slip events: the 2006 *M*7.5 slow slip events [red; (*7*)] and the *M*6.4 slow slip events [yellow; (*8*)]. The dark red patches indicate the intermittent slow transients during the 2006 slow slip event (*12*), highlighting the short slip duration compared to the 6-month event duration. (**C**) Schematic of the Guerrero subduction zone, with inset showing geographical location. The two LFE source regions are indicated by the blue and purple boxes. The red and orange patches correspond to areas where geodetically observed slow slip occurs.

We then explore in Fig. 1 how LFE amplitudes vary with the slow slip that is geodetically resolvable and dominates the tectonic slip cycle in Guerrero. Every 4 years, the regional GPS network captures a 6-month-long slow slip event that releases as much built-up tectonic stress as an *M*7.5 earthquake (*7*, *10*). Further downdip, smaller *M*6.4 slow slip events have previously been identified in the GPS record using recurring bursts of low-frequency seismicity as a guide (*8*, *11*). We observe the strongest LFE amplitudes in the updip source region, where LFE activity is most strongly modulated by slow slip (*6*). Notably, LFE amplitudes systematically increase during major slow slip events, implying that the LFE seismic moment rate ${\dot{M}}_{o}^{\text{seis}}$ tracks the geodetic moment rate ${\dot{M}}_{o}^{\text{geo}}$ of slow slip.

We capitalize on this observation by focusing on the 58 LFE sources within the updip source region in Guerrero (fig. S2) to estimate the average LFE ${\dot{M}}_{o}^{\text{seis}}$ during each geodetically constrained slow slip event in Fig. 1B. We measure ${\dot{M}}_{o}^{\text{seis}}$ as the median displacement amplitude of all of the LFEs during the slow slip event. The median LFE amplitude during the *M*6.4 slow slip events is 35% smaller than that of the major 6-month *M*7.5 slow slip event in 2006. The observation suggests that the average LFE seismic moment rate is lower during smaller magnitude slow slip events, and thus, a calibration between the geodetic moment rate and the seismic moment rate is reasonable.

### Geodetic moment rate of slow slip during large slow slip events

**Fig. 2:**
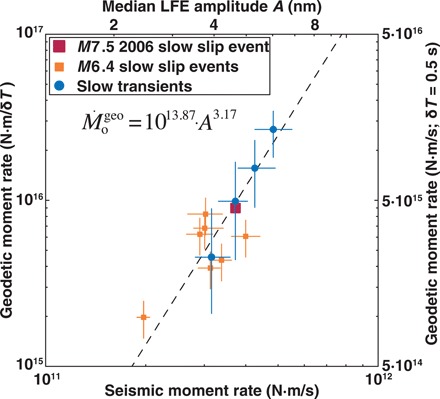
Geodetic slow slip moment rate, estimated from GPS surface displacements, scales with the seismic moment rate of slow slip, measured from the displacement amplitudes of LFEs. Units of δ*T* reflect the average duration of the aseismic pulses that drive LFE activity; we assume δ*T* = 0.5 s (*14*, *15*) for the right *y* axis. The squares indicate the moment rates of geodetically observed slow slip events. The blue circles reflect 1-day slow transients, representing the average moment rate for a given range of LFE amplitudes (fig. S1). See Materials and Methods for a discussion of error bars.

**Fig. 3:**
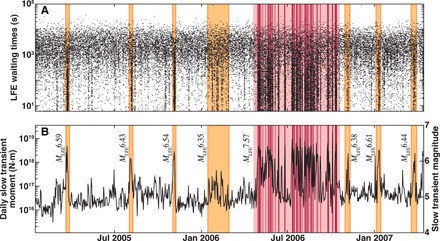
Daily tectonic release of slow slip along the Guerrero subduction interface, as tracked by LFEs. The yellow and red patches respectively highlight the geodetically observed small *M*6.4 and large *M*7.5 slow slip events (Fig. 1). The dark red patches indicate the intermittent slow transients during the 2006 slow slip event (*12*). (**A**) Waiting times between successive LFEs in the updip LFE source region highlight the clustered seismicity that accompanies slow slip (*29*). (**B**) Evolution of daily slow transient magnitudes, estimated with the moment rate scaling shown in Fig. 2. The small slow slip events are made up of a single group of slow transients, while the 2006 *M*7.5 slow slip event is composed of a complex cluster of these transients.

With estimates of the average seismic moment rate ${\dot{M}}_{o}^{\text{seis}}$ during each geodetically observed slow slip event, we now measure the corresponding average geodetic moment rates ${\dot{M}}_{o}^{\text{geo}}$ (Fig. 2). We start with the geodetic estimates of slow slip moment $M_{o}^{\text{geo}}$ (*7*, *8*, *12*). Because we need a moment rate, rather than moment, we must divide by a duration. The GPS record in Fig. 1B suggests that the total event duration of the 2006 *M*7.5 slow slip event is 6 months. However, recent work has demonstrated that when there is little to no LFE activity during slow slip, the geodetic record reflects tectonic loading and not slip (*12*). The episodic LFE activity during the 2006 slow slip event (Fig. 3A) suggests that at most 30% of the event duration involves active slip on the plate interface; the active slipping time is likely much shorter given the coarse daily sampling of the GPS record. We thus seek a proxy for slip duration that is restricted to the time period during which slip occurs.

Because slow slip is most prominent in the geodetic record during LFE activity (*12*, *13*), we suggest that the duration of LFE activity is a good proxy for the active slow slip period. We thus use the number of LFE per day *N*_LFE_ as a proxy for slow slip duration. We estimate ${\dot{M}}_{o}^{\text{geo}}$ as $M_{o}^{\text{geo}}/(N_{\text{LFE}}\delta T)$, where δ*T* represents the average duration of the aseismic slip pulses that drive LFE activity. That is, δ*T* is a quantization of the duration of slow slip that accumulates over all LFEs to estimate the total slip duration *T* on each day. The simplest interpretation is that the duration of the driving aseismic slip is the same as the source duration of LFEs, which previous studies have estimated at 0.2 to 0.7 s (*14*, *15*). That said, we maintain generality here by keeping δ*T* as a variable throughout the calculations while still providing results with physical units using a typical value of δ*T* = 0.5 s to highlight the approximate scale of the observations. We now have a proxy for geodetic moment rate that can be compared to the seismic moment rate.

### Identification and measurement of additional slow slip events

The power of combining the LFEs with the geodetic data is in the ability to detect otherwise invisible slow slip events. We therefore gather together GPS data on days with similar median LFE amplitudes to find additional events. GPS position solutions are daily; therefore, higher temporal resolution is not possible for this dataset. We bin by amplitude as finely as possible while minimizing overlap and maintaining a similar number of data points in each bin (see Materials and Methods). Each amplitude bin corresponds to a slow transient that generates a given range of LFE moment rates (fig. S1). The surface displacement rate of each newly identified slow transient is measured as the velocity of the cumulative sum of the binned GPS displacements, following the method of Frank (*13*). The geodetic moment $M_{o}^{\text{geo}}$ is then estimated as the moment of the fault dislocation that generates 1 day of displacement at the estimated surface displacement rate (*16*). As above, these geodetic moments are then converted to moment rate by normalizing by their slip duration, estimated as the mean number of LFEs per day in each amplitude bin multiplied by δ*T*. We measure the seismic moment rate ${\dot{M}}_{o}^{\text{seis}}$ of each slow transient as the median LFE amplitude in each amplitude bin.

### Calibration of seismic to geodetic moment rates

The resulting moment rate estimates in Fig. 2 appear to follow a power-law trend of the form

$${\dot{M}}_{o}^{\text{geo}}=\alpha A^{\beta }\propto {\left({\dot{M}}_{o}^{\text{seis}}\right)}^{\beta }$$ (1)

where *A* is the median LFE displacement amplitude, and α and β are scaling coefficients. We estimate the best-fitting power law with an errors-in-variable linear regression; the best-fit values of α and β are 10^13.87 ± 0.19^ and 3.17 ± 0.29, where *A* is measured in nm and ${\dot{M}}_{o}^{\text{geo}}$ is measured in N-m/δ*T*. If δ*T* is approximately 0.5 s as estimated by previous studies (*14*, *15*), the seismic moment rate in the study range is three to four orders of magnitude smaller than the geodetic moment rate. A calibration between the geodetic moment rate of slow slip and the seismic moment rate of LFEs has been achieved.

We note that the slow transients derived from the LFE analysis are critical to this calibration. If only the eight geodetically observed slow slip events in Fig. 2 (orange and red symbols) are considered, then the relationship between seismic and geodetic moment rate is not obvious. The largest *M*7.5 slow slip exhibits higher than average geodetic and seismic moment rates. The small slow slip event of February 2006 displays the lowest moment rates; this slow slip lasts five times longer than the other six *M*6.4 events and exhibits a flat GPS signature alongside small LFE amplitudes (Fig. 1). However, most geodetically observed slow slip events are tightly clustered around fairly similar moment rates, and LFEs are necessary to identify a fuller range of dynamics.

## DISCUSSION

### Daily record of slow slip inferred from LFEs

Now that we have a well-calibrated scaling of seismic and geodetic moment rates, we can use this relationship to estimate the slow transients that drive LFE activity during every day of the seismic record. We do this by converting the daily median LFE amplitude into geodetic moment rate and then by multiplying the individual event slip duration to find the total daily slip (*T* = *N*_LFE_δ*T*). Figure 3B shows the resulting catalog of slow transients. We observe a *M* > 4.5 slow earthquake every day along the subduction interface beneath Guerrero; at least 6 × 10^15^ N-m of slow slip is released by the plate interface each day. The total moment released over the 2.5-year study period is equivalent to an *M*7.64 earthquake. Previous studies documented direct (*13*, *17*) and indirect (*18*) evidence that slow slip occurs more often than is obvious in the geodetic record. Here, we demonstrate that slow slip occurs on subdaily time scales, and we are able to measure the moment released every day.

Subdaily slow transients have been sought before by using time series of repeating earthquakes as proxies for fault slip (*19*–*21*). The repeating earthquake slip proxy depends strongly on an assumed interaction between the seismic and aseismic fault processes. We do not need to rely on that assumption, because the seismic-geodetic calibration allows us to measure the daily slow slip moment for the first time.

The resulting calibration is similar in spirit to the calibration between tremor and geodetic moment rate suggested by Aguiar *et al*. (*22*). This previous study, however, used tremor duration rather than LFE amplitude and focused only on large, relatively long duration events. That work inferred a constant moment rate for geodetic slip, which is inconsistent with the empirical scaling of Fig. 2. With the help of LFEs, we are able to sample high moment rates during slow slip events that would otherwise be lost to averaging over a highly variable, intermittent slip process.

The large geodetically observed slow slip events in Fig. 3 appear to be made up of many shorter ~*M*6 transients. Most of the *M*6.4 slow slip events appear as single sequences of these large slow transients. The 2006 *M*7.5 slow slip event is an intermittent sequence of *M*6 slow transients (*12*). The characteristic size of these slow transients echoes the recently reported characteristic rates of tectonic tremor activity during both major and small slow slip events (*23*). The magnitude-frequency distribution of slow transients is not well represented by an earthquake-like power law and rather appears to have a characteristic size (fig. S4). However, the LFE amplitudes in isolation do follow a power law (fig. S1).

### Moment scaling of daily slow transients

**Fig. 4:**
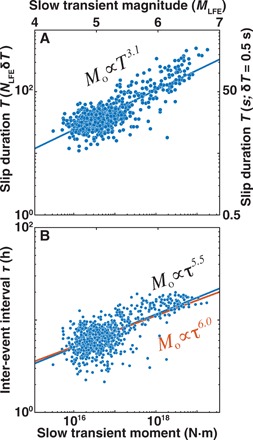
Moment scaling of slow transients in Guerrero as inferred from LFEs. (**A**) Slow transient moment *M*_o_ scales with the slip duration *T*^3^ (blue line), where duration is the number of LFEs multiplied by the average aseismic pulse duration of δ*T*; we assume δ*T* = 0.5 s (*14*, *15*) for the right *y* axis. The moment-duration scaling is the same as for ordinary earthquakes. (**B**) Slow transient moment scales with the inter-event interval τ to the power of 5.5 (blue line). The moment–inter-event interval scaling is approximately the same as for repeating earthquakes (orange line).

We now go further to examine the systematics of the newly discovered events. Past studies of slow earthquakes have suggested that their slip dynamics are fundamentally different than those of earthquakes (*24*). In previous work, a constant apparent moment rate implied that slow earthquake moment *M*_o_ scales linearly with duration *T* (*25*). However, Fig. 4 shows that slow transient moment *M*_o_ scales with the slip duration *T* cubed. This slow transient moment-duration scaling is the same scaling relationship observed for earthquakes (*26*), implying that slow slip and earthquakes are more similar than previously thought. We speculate that the previously reported *M*_o_ ∝ *T* scaling (*24*) reflects the interaction of two distinct processes: the slow rupture process and the clustering process that links disparate slow transients into a large slow slip event (*12*). GPS observations are unable to provide the necessary resolution to distinguish between these two mechanisms, and capture the combination of both processes. Once we strip away this clustering process from slow slip events to examine how moment scales with slip duration, slow slip is revealed to follow the same scaling as fast earthquake slip. We can only speculate on the physical significance of the exponent of 3 in Eq. 1, and it remains to be seen whether it varies from one plate boundary to the next.

At least one additional systematic is in the data. We define a proxy for the average slow transient inter-event interval τ based on the longest waiting times between LFEs each day; this quantity represents the amount of time per day that slow slip is not happening. We estimate τ as the sum of the 10% longest LFE waiting times. This τ is similar to a recurrence time, but we prefer the more general term “inter-event time” to avoid any implication of periodic behavior. Figure 4 shows that slow transient moment increases with the inter-event time and scales with the same exponent of 6 as previously observed for repeating earthquakes (*19*, *27*). The observation suggests a commonality stemming from both processes involving seismic asperities surrounded by aseismic ruptures with varying degrees of partitioning between seismic and aseismic slip.

### Conclusions

We have demonstrated here that slow transients occur every day by constraining the seismic-geodetic moment rate scaling of slow slip, linking the dynamics of low-frequency seismicity to the geodetic signature of the driving aseismic slip. The existence of these minutes-long daily transients fills in an observational gap, suggesting that the spectrum of slip is continuous (*2*, *28*). Accounting for the clustered and intermittent rupture process of slow slip, we find that the moment-duration scaling of these daily slow transients is the same as ordinary earthquake scaling, and the moment-recurrence scaling follows the same relationship as repeating earthquakes. In aggregate, our observations suggest that slow slip on a plate interface is similar to ordinary earthquakes, and a broad continuum of transient slip governed by one set of dynamics may explain both phenomena.

## MATERIALS AND METHODS

### Estimating LFE displacement amplitudes and seismic moment rate

We measured the displacement amplitude *A* of all observed LFE *S* waves on each horizontal component of the 10 recording seismic stations (*6*). First, we measured the RMS amplitude in a 6-s window centered on the LFE *S* wave in the frequency band of 1 to 2 Hz. This window duration matches the average length of LFE *S* waves in Guerrero, and the frequency band contains the highest signal-to-noise ratios in these events (*6*). We corrected for geometric spreading of the form *r*^−1^, where *r* is the source-receiver distance and then averaged the resulting, corrected amplitudes over all stations and components.

Displacement amplitude is directly proportional to seismic moment rate (*9*)

$$A\approx \frac{R{\dot{M}}_{o}}{{4\pi r\rho \beta }^{3}}$$ (2)

where *R* is the *S*-wave radiation pattern, *r* is the distance from the source to the closest station (42.5 km), ρ is the average density of the medium over the source-receiver path (assumed to be 2850 kg/m^3^), and β is the average *S*-wave velocity of the source-receiver path (assumed to be 3.75 km/s). We therefore converted LFE amplitudes into seismic moment rates using Eq. 2. During this process, we neglected the radiation pattern *R*, as all LFEs were in a similar geometry relative to the stations. To confirm that the largest measured amplitudes are not biased by other seismic signals, we plotted the evolution of the 5% largest LFEs in fig. S5. We observed that their activity was synchronized with geodetically observed slow slip, with the largest amplitudes (and thus moment rates) occurring as expected during the 2006 *M*7.5 slow slip event. The final resulting distribution of amplitudes is shown in fig. S1.

### LFE amplitude binning to derive slow transients

We selected the LFE amplitude bins to contain at least 30 data points per bin while minimizing overlap. The four bins chosen for Fig. 2, from lowest to highest moment rates, contained 52, 41, 47, and 35 days of data. Following the method of Frank (*13*), the GPS displacements in each bin were cumulatively summed, and the average displacement per day was estimated as the slope of the best-fit linear trend.

To investigate the sensitivity of the results to binning, we performed the same analysis as described in the main text with three rather than four amplitude bins. We used the same criteria described above to select three amplitude bins, except that we required a minimum of 40 data points in each bin. We observed a power-law relationship between seismic and geodetic moment rates in fig. S3 with scaling coefficients of α = 10^13.89 ± 0.26^ and β = 3.15 ± 0.40. This trend is similar to the relationship constrained in Fig. 2.

### Error estimation in seismic-geodetic moment rate scaling

The geodetic moment rate errors in Fig. 2 for the geodetically observed slow slip events reflect the observational surface displacement errors. These errors reflect the uncertainty of the estimated static displacement offsets that control the inversion for the distribution of slow slip on the interface. The displacement error for the 2006 *M*7.5 slow slip event represents ~10% of the estimated moment (*7*). The average displacement error for the seven *M*6.4 slow slip events represents ~25% of the estimated moment (*8*). The uncertainties of the amplitude-derived slow transients were determined with a jackknife analysis. We performed a 10% jackknife of the LFEs in each amplitude bin, determined the average surface displacement per day following the method of Frank (*13*), and estimated the moment as the corresponding fault dislocation (*16*). We estimated the geodetic moment rate error of each slow transient as the 10th and 90th percentiles of the resulting jackknifed distribution of estimated moments.

The seismic moment rate errors were estimated with a similar jackknife analysis. We performed a 10% jackknife of the LFEs in each slow slip event or slow transient and recomputed the median LFE amplitude. We estimated the error bars as the 10th to 90th percentiles of the resulting jackknifed distribution of median LFE amplitudes.

The errors in the estimated power-law coefficients α and β were determined by performing a one-sample jackknife of the 12 moment rate data points (8 geodetically constrained slow slip events and 4 amplitude-derived slow transients) and then fitting a new power-law relationship to each jackknifed resampling. We estimated the errors of α and β as the standard deviation of the jackknifed distribution of coefficients.

## Supplementary Material

http://advances.sciencemag.org/cgi/content/full/5/10/eaaw9386/DC1

Download PDF

Daily measurement of slow slip from low-frequency earthquakes is consistent with ordinary earthquake scaling

## SUPPLEMENTARY MATERIALS

Supplementary material for this article is available at http://advances.sciencemag.org/cgi/content/full/5/10/eaaw9386/DC1 Fig. S1. Distribution and evolution of LFE displacement amplitudes in Guerrero. Fig. S2. Tectonic context of the subduction zone underneath Guerrero, Mexico. Fig. S3. Alternative number of slow transients to constrain the seismic to geodetic moment rate relationship shown in Fig. 2. Fig. S4. Distribution of slow transient magnitudes. Fig. S5. Daily count of the 5% largest LFEs (2337 events); the plotted amplitudes are *>*12.4 nm. Reference (*30*)
