# Supplementary material for: Daily measurement of slow slip from low-frequency earthquakes is consistent with ordinary earthquake scaling
Source: Sci Adv. 2019 Oct 2;5(10):eaaw9386. doi: 10.1126/sciadv.aaw9386 (PMC6774729; doi:10.1126/sciadv.aaw9386)
Supplement: http://advances.sciencemag.org/cgi/content/full/5/10/eaaw9386/DC1 [file supp_5_10_eaaw9386__index.html]

Science Advances | Science AdvancesAAASSearchScience AdvancesMenu

## Supplementary Materials

**This PDF file includes:**

- Fig. S1. Distribution and evolution of LFE displacement amplitudes in Guerrero.
- Fig. S2. Tectonic context of the subduction zone underneath Guerrero, Mexico.
- Fig. S3. Alternative number of slow transients to constrain the seismic to geodetic moment rate relationship shown in Fig. 2.
- Fig. S4. Distribution of slow transient magnitudes.
- Fig. S5. Daily count of the 5% largest LFEs (2337 events); the plotted amplitudes are *>*12.4 nm.
- Reference (*30*)

Download PDF

**Files in this Data Supplement:**

- Adobe PDF - aaw9386\_SM.pdf
