# Supplementary material for: Daily measurement of slow slip from low-frequency earthquakes is consistent with ordinary earthquake scaling
Source: Sci Adv. 2019 Oct 2;5(10):eaaw9386. doi: 10.1126/sciadv.aaw9386 (PMC6774729; doi:10.1126/sciadv.aaw9386)
Supplement: Download PDF [file aaw9386_SM.pdf]

Science Advances
AAAS

advances.sciencemag.org/cgi/content/full/5/10/eaaw9386/DC1

Supplementary Materials for

Daily measurement of slow slip from low-frequency earthquakes is consistent with ordinary earthquake scaling

William B. Frank* and Emily E. Brodsky

*Corresponding author. Email: wbfrank@usc.edu

Published 2 October 2019, *Sci. Adv.* **5**, eaaw9386 (2019)
DOI: 10.1126/sciadv.aaw9386

This PDF file includes:

Fig. S1. Distribution and evolution of LFE displacement amplitudes in Guerrero.
Fig. S2. Tectonic context of the subduction zone underneath Guerrero, Mexico.
Fig. S3. Alternative number of slow transients to constrain the seismic to geodetic moment rate relationship shown in Fig. 2.
Fig. S4. Distribution of slow transient magnitudes.
Fig. S5. Daily count of the 5% largest LFEs (2337 events); the plotted amplitudes are >12.4 nm.
Reference (*30*)

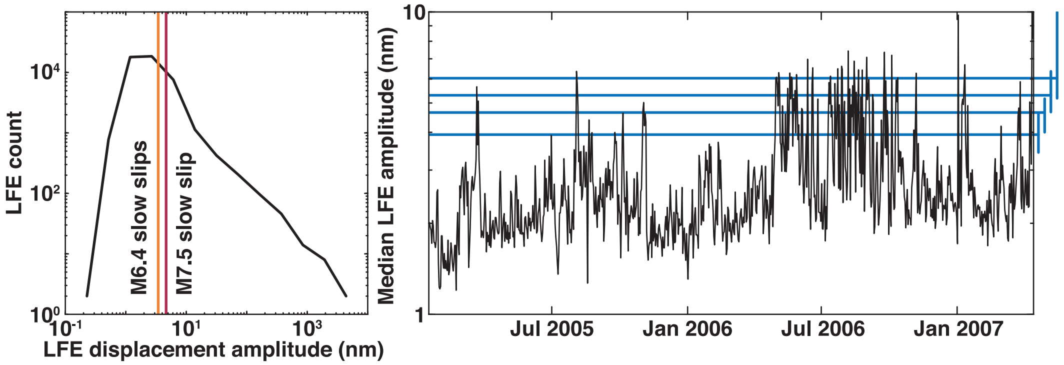

Fig. S1. Distribution and evolution of LFE displacement amplitudes in Guerrero. (a) Histogram of the 46,732 LFE amplitudes within the updip LFE source region. The vertical lines highlight that the median LFE amplitude during the *M*7.5 slow slip event (red) is 35% greater than the median amplitude during the seven *M*6.4 slow slip events (yellow). (b) Daily evolution of the median LFE amplitude within the updip LFE source region (Fig. 1). The blue lines indicate the median amplitude of each amplitude bin used to identify slow transients in Fig. 2. The vertical bars to the right show the full range of each bin.

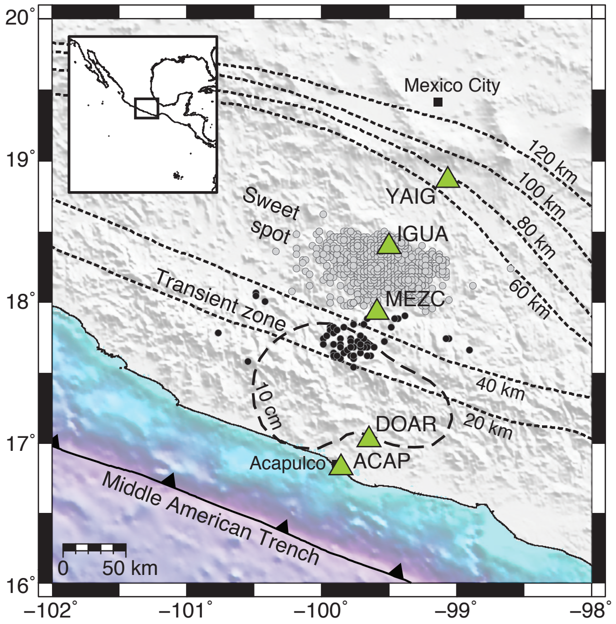

Fig. S2. Tectonic context of the subduction zone underneath Guerrero, Mexico. A large slow slip event in 2006 recorded by GPS (green triangles) accumulated more than 10 cm of slip (thick dashed contour) updip of low-frequency earthquake (LFE) sources (6) (black points: analyzed 58 updip sources; gray points: downdip sources). Depth contours of the subduction interface (30) are shown as thin dashed lines. Figure is directly reproduced from ref. 12 and included here to provide complete documentation of the dataset.

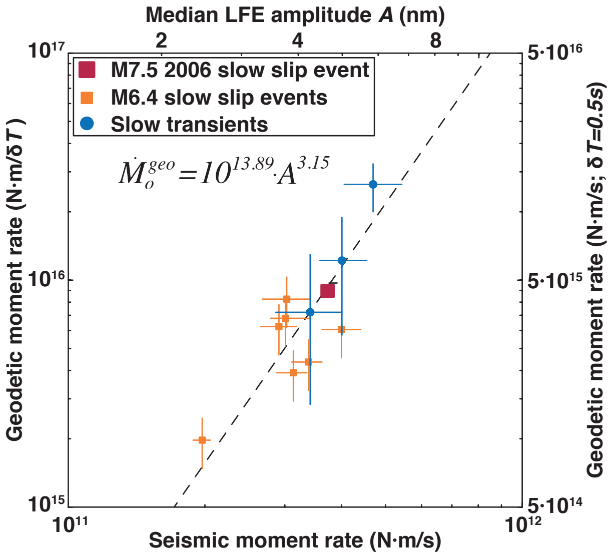

Fig. S3. Alternative number of slow transients to constrain the seismic to geodetic moment rate relationship shown in Fig. 2. Units of δT reflect the average duration of the aseismic pulses that drive LFE activity. The squares indicate the moment rates of geodetically-observed slow slip events. The blue circles reflect one-day slow transients, representing the average moment rate for a given range of LFE amplitudes (fig. S1).

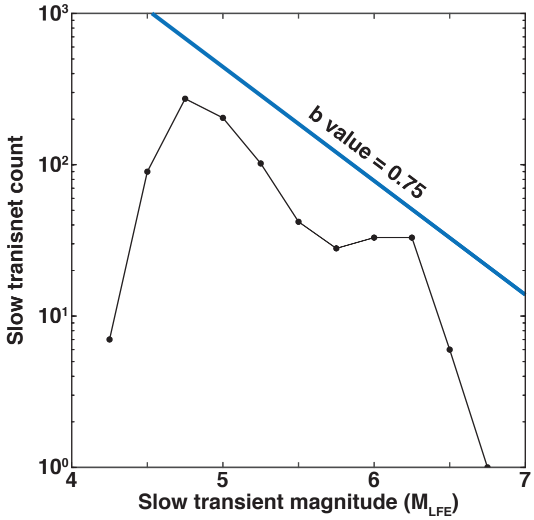

Fig. S4. Distribution of slow transient magnitudes. The best-fit power law (blue line) does not represent well the slow transient distribution, whose defining feature is characteristic M6 events.

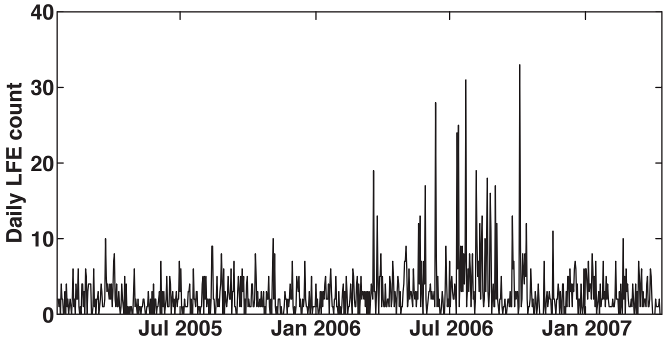

Fig. S5. Daily count of the 5% largest LFEs (2337 events); the plotted amplitudes are >12.4 nm. The evolution of the largest low-frequency earthquakes tracks well geodetically observed slow slip events with the largest amplitudes (and thus moment rates) occurring in mid-2006 during the M7.5 slow slip event (Fig. 1).
